# Gene evolutionary trajectories in *Mycobacterium tuberculosis* reveal temporal signs of selection

**DOI:** 10.1073/pnas.2113600119

**Published:** 2022-04-22

**Authors:** Álvaro Chiner-Oms, Mariana G. López, Miguel Moreno-Molina, Victoria Furió, Iñaki Comas

**Affiliations:** ^a^Instituto de Biomedicina de Valencia (IBV-CSIC), Valencia, 46010, Spain;; ^b^CIBER en Epidemiología y Salud Pública, Valencia, Spain

**Keywords:** *Mycobacterium tuberculosis* complex, genomics, pathogen evolution

## Abstract

Genetic differences between different *Mycobacterium tuberculosis* complex (MTBC) strains determine their ability to transmit within different host populations, their latency times, and their drug resistance profiles. Said differences usually emerge through de novo mutations and are maintained or discarded by the balance of evolutionary forces. Using a dataset of ∼5,000 strains representing global MTBC diversity, we determined the past and present selective forces that have shaped the current variability observed in the pathogen population. We identified regions that have evolved under changing types of selection since the time of the MTBC common ancestor. Our approach highlighted striking differences in the genome regions relevant for host–pathogen interaction and, in particular, suggested an adaptive role for the sensor protein of two-component systems. In addition, we applied our approach to successfully identify potential determinants of resistance to drugs administered as second-line tuberculosis treatments.

## Significance

Previous attempts to identify the action of natural selection in the *Mycobacterium tuberculosis* complex (MTBC) were limited by sample size and averaging across time and lineages. We investigate changes in selective pressures across time for every single gene of the MTBC. We developed a methodology to analyze temporal signals of selection in a large dataset (∼5,000 complete genomes) and showed that 1) almost half of the genes seem to have been under positive selection at some point in time; 2) experimentally confirmed epitopes tend to accumulate more mutations in deeper branches than in external branches; and 3) temporal signals identify genes that were conserved in the past but under positive selection in the present, suggesting ongoing adaptation to the host.

The *Mycobacterium tuberculosis* complex (MTBC) is a genetically monomorphic group of bacteria (1, 2) whose members cause tuberculosis in humans and animals. The MTBC comprises both human-associated (L1, L2, L3, L4, L5, L6, L7, L8, and L9) and animal-associated (A1, A2, A3, and A4) clades (3–7). Due to the absence of horizontal gene transfer, plasmids, and measurable recombination among strains and other species (8–10), chromosomal mutations represent the source of MTBC genetic diversity. The maximum genetic distance between any two MTBC strains is around 2,500 single-nucleotide polymorphisms (SNPs). Strikingly, studies have highlighted large phenotypic differences between strains involving traits like gene expression, drug resistance, transmissibility, and immune response, despite this limited variation. In some cases, the mutations driving phenotypic differences have been identified—for example, nonsynonymous variants in genes, such as *rpoB*, *katG*, or *gyrA*, cause drug-resistant phenotypes (11–13). Furthermore, single mutations in regulatory elements can induce alterations to downstream gene expression, which can foster differential virulence characteristics (14, 15). Finally, specific gene mutations may affect transmission (9), host tropism within the complex (16), and the host immune response (17). However, many of the genomic determinants of these phenotypes remain elusive, despite robust evidence that they are driven by genetic differences between strains (18, 19).

Several types of evolutionary forces play crucial roles in the fixation of mutations in bacterial populations. Previous research has provided evidence for the ongoing positive selection of specific genes and regions (9, 20–23), while other studies have reported ongoing purifying selection of specific genomic regions, especially in epitopes and essential genes (24). Additionally, there exists some evidence that genetic drift may have significant functional and evolutionary consequences (25).

Detecting selection in MTBC at the genome-wide level remains a challenging task due to limited genetic diversity. The significant accumulation of nonsynonymous substitutions has been previously used to characterize patterns of mutation accumulation in large categories of genes (24, 26); however, these studies employed a limited number of strains. Of note, the number of MTBC sequences has undergone a recent and rapid expansion, with studies involving hundreds to thousands of strains. The large number of available sequences has allowed, for example, the estimation of the ratio of nonsynonymous to synonymous substitutions (dN/dS) signatures in more than 10,000 strains (27), thereby allowing the identification of targets of selection with some probably related to host–pathogen interactions. Host–pathogen interaction signals are specially challenging as they are likely obscured by the force exerted by antimicrobial therapies. Weaker signals are also expected in genes related to second-line drugs related to the relative underuse of related treatments and the low abundance of associated resistant strains in genome databases (28).

We reasoned that to detect signs of selection, we should focus on when and/or where they occurred in the phylogenetic tree instead of averaging signs across the phylogeny. In this study, we developed a methodology to study temporal signs of selection in MTBC genes and identified positive selection in a larger number of genes than previously described. This allowed the identification of past and currently unknown players in the MTBC evolution, particularly two-component systems (2CSs), related to host adaptation and second-line drug resistance. This methodology can be applied to other tuberculosis settings to explore signs of selection associated with changing selective pressures and could be extremely useful to unravel hidden details in the evolution of other human pathogens.

## Results

### Dataset Preparation.

We downloaded all samples described in Brites *et al.* (4), Coll *et al.* (29), Stucki *et al.* (30), Guerra-Assunção *et al.* (31), Zignol *et al.* (32), Bos *et al.* (33), Ates *et al.* (34), Comas *et al.* (10, 35), Borrell *et al.* (36), and Cancino-Muñoz *et al.* (37) and obtained whole-genome sequencing data from 9,240 samples comprising the primary human- and animal-adapted MTBC lineages. We mapped FASTQ files for each sample against the inferred ancestor of the MTBC and extracted genomic variants (*Materials and Methods*), from which we derived a multiple sequence alignment and a phylogeny. The size of the phylogeny and the multifasta file obtained made some parts of the planned subsequent computational analyses unaffordable. Hence, we used Treemmer to prune the tree down to 4,958 leaves (Dataset S1) while maintaining 95% of the original genetic diversity. With this final set of selected samples, we reconstructed a multiple sequence alignment and a phylogeny (*SI Appendix*, Fig. S1*A*).

We mapped each genomic variant to the inferred phylogeny using PAUP (Phylogenetic Analysis Using Parsimony). This step provides information regarding the branch in which every mutation appeared, which allows the identification of homoplastic variants—those that appeared multiple times in different branches of the phylogeny—and the relative “age” of every mutation calculated as the node to root genetic distance.

### Scars of Past Selection and Drift in Almost Half of the MTBC Genome.

**Fig. 1.**
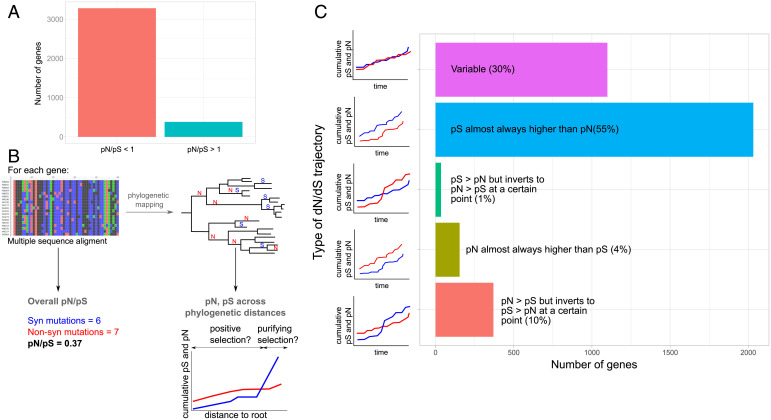
Gene-by-gene calculation of pN/pS over phylogenetic time. (*A*) Bar plot showing the number of genes currently displaying a pN/pS greater than one and a pN/pS less than one. (*B*) From the alignment, we inferred the current pN/pS; however, when mapping different mutations onto the phylogeny, we inferred how the pN and pS rates changed over time. S and N symbols on the phylogeny correspond to potential synonymous and nonsynonymous variants. (*C*) Five categories grouping studied genes according to their trajectories.

As a first step, we calculated the ratio of the relative abundance of nonsynonymous and synonymous polymorphisms (pN/pS) values for genes that possessed up to 10 identified variants (*n* = 3,690). A previous study stated a mean pN/pS value for the complete MTBC genome considerably under one (38). In agreement with this result, we found that 90% of the genes evaluated possess a pN/pS value less than one (Fig. 1*A*) (pN/pS interquartile range [IQR] = 0.477 to 0.804), suggesting ongoing evolution under purifying selection. A high pN/pS may reflect the recent origin of the MTBC given the time-dependent nature of the accumulation of nonsynonymous variants (39).

Of note, the pN/pS value for a gene results from the pN and pS values calculated with all gene mutations found across the phylogeny (what we term the “overall pN/pS” in Fig. 1*B*). This value does not reflect changes in selective pressures over time and lineages as the pathogen has potentially faced different environmental “challenges.” As we estimated the genetic distance to the root for each mutation as a relative measure of time, we calculated temporal trajectories for pN/pS for each gene during MTBC evolution (Fig. 1*B* and *Materials and Methods*). This allowed us to detect, on the one hand, genes that have been subjected to directional selection (either positive or negative) across all their phylogenetic history and on the other hand, genes that have suffered from changing types of selection. So, we classified all genes according to their pN and pS trajectories over time into five different categories (Fig. 1*C*, Dataset S2, and *SI Appendix*, Fig. S2*A*): 1) pS almost always higher than pN (*n* = 2,032); 2) pN almost always higher than pS (*n* = 154); 3) pS > pN but inverts to pN > pS at a certain point (*n* = 35); 4) pN > pS but inverts to pS > pN at a certain point (*n* = 370); and 5) complex pN and pS trajectories with multiple cross-points, which do not support proper categorization (*n* = 1,099).

To check if our classification is suitable, we have performed two different statistical tests that we have called the “stability of trajectories test” and the “natural selection test.” Regarding the stability of trajectories test, if our classification reflects differences in the selection pattern over time, we expect that those genes with stable trajectories (“always higher”/“always lower”) will have accumulated low variation in pN/pS when pooling time points. Conversely, we expect changing trajectories to display high variation between time points (*Materials and Methods* and *SI Appendix*, Fig. S2*B*). As predicted, we failed to observe significant differences in pN/pS variation in genes belonging to the “pN almost always higher” and “pS almost always higher” categories if we compared them against the null hypothesis of no change of pN/pS over time (*z* scores of −0.47 and −0.13, respectively). In both cases, the pN/pS cumulative variation has a value around zero. However, categories with changing trajectories displayed significant differences (*z* scores of 1.92 and −1.70) against the same null hypothesis. Next, with the natural selection test, we tested if those genes with pN/pS stable over time and either higher than one or lower than one likely reflect selection. We compared, for each gene in these categories, the mean pN/pS values across time in log scale against the null hypothesis provided by the mean trajectory of pN/pS across time for the complete genome. The mean trajectory across genes converges towards a value close to 0.6, reflecting the action of purifying selection (*SI Appendix*, Fig. S3). We expected that the category associated with purifying selection was not significant when compared with the mean trajectory of the genome which already has a pN/pS below 1 (0.64). On the contrary, the category associated with pN > pS was expected to be significantly different, reflecting the action of likely positive selection. In agreement, we obtained the following *z* scores: 0.82 (*P* value = 0.7) for pS always higher than pN and −2.55 (*P* value = 0.005) for pN always higher than pS.

In summary and in contrast with the observation that 90% of genes possess an overall pN/pS less than one, only 55% of genes (*n* = 2,032) maintained a pN/pS value below a value of one since divergence from the MTBC common ancestor. This set of 2,032 genes is overrepresented for experimentally confirmed essential genes in both *in vivo* and *in vitro* conditions (χ^2^ test, *P* value = 0.003 and *P* value < 2.2E-16, respectively). In contrast, 45% of the genes (*n* = 1,658), mainly those initially classified as being under purifying selection, may have faced other types of selective pressures or genetic drift.

These results suggest that many genes have been subjected to periods of nonsynonymous substitution accumulation. Distinguishing between genetic drift and positive selection at a particular time point remains challenging. We expect founder effects to play a crucial role during the early evolution of MTBC, and they may drive a number of the unstable trajectories observed. However, given that MTBC is clonal, positive selection and genetic drift are both expected to have a functional impact. Our analysis identifies a set of genes that shows a pN/pS greater than one near the root but that changed to pN/pS less than one near the leaves (*n* = 370), suggesting that selection and/or founder effects favored the fixation of nonsynonymous mutations at early times but that the amino acid sequence remained conserved at later times. We found that this gene category was enriched for conserved hypotheticals (Fisher test, *P* value = 0.02) and protein and peptide secretion (Fisher test, *P* value = 0.05). Intriguingly, we also discovered that certain genes that fell into this category encode known MTBC epitopes (which we will explore below). Of particular note is the presence of 154 genes almost always exhibiting a pN higher than pS. This gene category is enriched for nonessential *in vitro* genes (χ^2^ test, *P* value = 0.005) from three main categories: antibiotic production and resistance (Fisher test, *P* value = 0.02, further explored below), conserved hypotheticals (Fisher test, *P* value = 0.02), and unknown functions (Fisher test, *P* value = 0.03). The mix of genes with a clearly identified function and hypothetical genes suggests that, in some cases, positive selection has been acting through the evolutionary story of some genes, while others are likely under genetic drift.

### Evolutionary Trajectories Identify Sensor Proteins of 2CSs under Positive Selection.

An increasing rate of nonsynonymous mutations as we move toward the tips of the phylogeny is compatible with a change in the action of natural selection but also with unpurged transitory polymorphisms arising within transmission clusters or during within-host evolution. However, due to our sampling scheme that maximizes global diversity, this last scenario will only apply to a few cases where the trajectory changes at the very tips of the phylogeny. In addition, these nonsynonymous mutations appear all across the phylogeny, as expected for an MTBC-wide evolutionary phenomenon rather than for specific intrahost selection or local epidemiological patterns. To distinguish between the two possibilities (natural selection vs. transitory polymorphisms), we examined the group of genes with a pS > pN in the internal branches but a pN > pS near the leaves (*n* = 58) (Dataset S2). Antibiotic resistance genes represent a clear instance of recent positive selection, and we hypothesized that their initial trajectory should reflect conservation of gene function, as they usually perform relevant biological functions and only recently started to diversify due to antibiotic selective pressure. Encouragingly, data for the antimicrobial resistance genes, such as *rpoB*, *katG*, *embB*, *gidB*, and *rpsL*, supported this hypothesis. The distance to the root value is a measure of relative time. Thus, we wondered if we would be able to date the phylogeny and the mutations to unravel the exact moment at which the selective forces started to act in these genes. We have used Beast 2.6 to infer the age at which the mutations have occurred for some of these drug resistance (DR) genes. Later, we recalculated the pN/pS trajectories for these genes based on the inferred age instead of the distance to root values and saw the moment at which the pN/pS surpasses one, meaning that positive selection started to act. This point was placed at 72.92 y ago for *gidB* (streptomycin), 26.9 y ago for *embB* (ethambutol, 1966), and 63.55 y ago for *rpoB* (rifampicin, 1972). These results suggest that our approach possesses sufficient sensitivity to detect recent instances of positive selection. However, this dating approach has several limitations (*Discussion*).

Among those genes unrelated to antimicrobial resistance, we found several components of toxin–antitoxin systems, including *vapC29*, *vapB3*, *vapC35*, *vapB40*, *vapC22*, and *vapC47*, which are critical for the adaptation of bacteria to different stressful conditions. For example, VapC22 has a significant role in virulence and innate immune responses in particular (40). Other significant virulence regulators in MTBC are the 2CSs, which are critical players in extended transcriptional networks. 2CSs comprise a sensor protein coupled to a transcription factor; the sensor protein activates the transcription factor in response to a specific stimuli to trigger a regulatory cascade. We have previously described *phoR*, which encodes the sensor component of the PhoPR 2CS, as an important player in MTBC evolution (9) as illustrated by the high levels of accumulation of nonsynonymous variants over time. Our data show that *kdpD*, a gene that encodes the sensor component of the KdpDE 2CS, displays a similar pattern, with a pN/pS value that reached approximately two at some points during MTBC evolution. In both 2CSs, the genes encoding the regulatory protein (*phoP* and *kdpE*) display high conservation at the amino acid level, with the pS values consistently higher than the pN values. For the NarLS 2CS, both the regulatory protein (*narL*) and the sensor protein (*narS*) exhibit changing patterns toward recent positive selection; however, as for the other described 2CSs, the sensor domain of *narS* accumulates more nonsynonymous variants (Fisher test, *P* value = 0.036). We proposed previously (9) that subtle modifications of the PhoR sensor domain may be useful for fine tuning the PhoPR activity in response to the different microenvironments associated with differences between hosts. This is in accordance with the role of PhoPR as a major regulator of virulence and other key infection processes (14). As this pattern is observed in other 2CS sensor proteins, this may suggest that these mutations could allow MTBC strains to adapt to varying environments during host–pathogen interaction.

### Epitope Mutations Are Older and Show Divergent Evolutionary Trajectories Compared with the Rest of the Antigen.

Contrary to many other pathogens, the *M. tuberculosis* genome regions recognized by the host tend to be conserved, albeit with some exceptions (24, 41). Given our results revealing past “scars” of selection in MTBC genes, we analyzed the pN/pS trajectory of a total of 179 antigens harboring 1,556 epitopes (42). Specifically, we aimed to evaluate a hypothesis that epitope and nonepitope regions of the antigen experience different selective pressures and that the former most likely reflects interactions with the immune system, while the latter reflects the evolution of gene function.

**Fig. 2.**
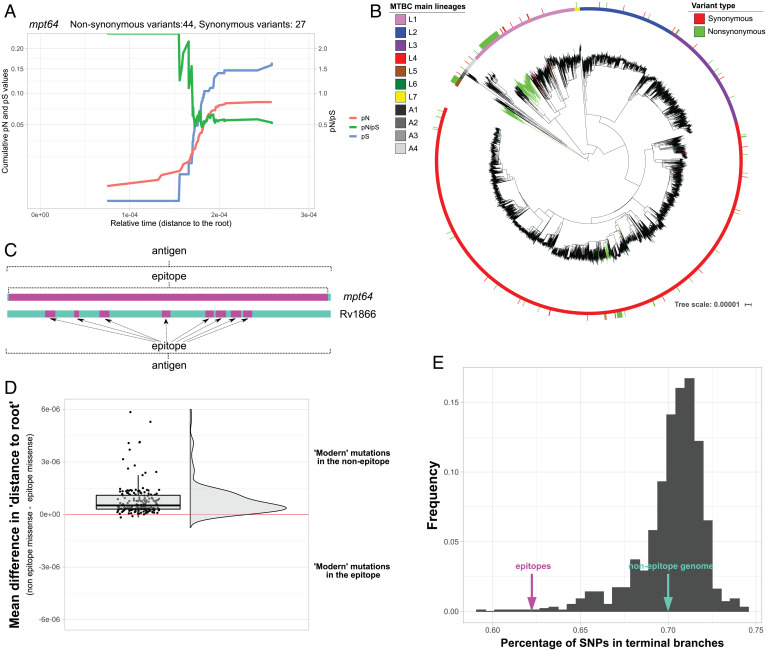
Specific antigenic genes show signs of early positive selection. (*A*) Cumulative pN, pS, and pN/pS trajectories over time for the *mpt64* gene (Rv1980c). The *x* axis represents the genetic distance of each node to the root. The left *y* axis represents the cumulative pN (red line) and pS (blue line) values. The right *y* axis represents the pN/pS. (*B*) Maximum likelihood MTBC phylogeny with mapped *mpt64* variants. The sticks in the outer circle mark the strains with variants identified (red, synonymous; green, nonsynonymous). Deep nonsynonymous mutations can be found in deep nodes of L1 and L5. (*C*) Some epitopes comprise the entire antigen (such as in *mpt64*), while in genes such as Rv1866, the epitope represents a small subset of regions embedded in the antigen. (*D*) Rain cloud plot of the mean differences in the distance (to root) value between the nonepitope and the epitope mutations for each antigen. (*E*) Distribution of SNPs found in terminal branches for 1,000 randomly selected sets of nonepitope fragments (gray bars). The percentage of SNPs observed in the epitopes differs from this distribution (∼62%, *z* score = −4,28; pink arrow), while the percentage of SNPs found in the rest of the genome remains similar to the distribution (∼70%; green arrow).

Our results revealed that ∼60% of the antigens analyzed exhibited a pN/pS value of less than one across phylogenetic history, providing evidence for their conservation since their diversification of the MTBC from a common ancestor (Dataset S3). Of note, a relevant proportion of antigens (11%) accumulated a high number of nonsynonymous variants in internal branches, which now appear to be conserved (Dataset S3). For example, the *mpt64* gene encodes for a known antigen employed in diagnostic tests. When mapping the genetic variants in the MTBC phylogeny, most nonsynonymous mutations map to the L5 ancestral branch in a large clade of the L1.2.2 sublineage and a group of L4.10 strains (Fig. 2 *A* and *B*). Other antigens, such as *eccD2*, Rv1866, *fadD21*, or Rv2575, exhibited a similar pattern. Apart from human-adapted clades, specific antigens accumulated nonsynonymous mutations in deep branches of the animal-adapted lineages, such as Rv2575 or *IlvB1*. This suggests that these antigens were under positive selection or genetic drift driven by founder effects when the MTBC diversified.

For another group of antigens (27%), the pN/pS value failed to show a definitive trajectory (Dataset S3). Specific antigens showed a pattern of pN/pS value of approximately one since the diversification of the MTBC from a common ancestor. This pattern could reflect two different causes: genetic drift or differential selective pressures in different MTBC clades/lineages, which could be masked when calculating a common pN/pS for all lineages. The second option is defined by an accumulation of nonsynonymous mutations in specific MTBC clades and synonymous mutations in other clades. As a result, the overall pN/pS value would be approximately one. We observed this scenario, for example, in the *lpqL*, *mce2A*, and *esxH* genes; in these cases, we found an elevated accumulation of nonsynonymous mutations in deep branches of the L1, L2, and *Mycobacterium africanum* lineages, although they are highly conserved in modern lineages. Other genes exhibited a similar pattern (Dataset S3), while others could have evolved under the effect of genetic drift.

In general, the evolution of antigens does not essentially differ from other genes in their respective functional categories. When we compared the trajectories of the antigens against such genes, we failed to encounter statistical differences between the distributions (Fisher test, Benjamini-Hochberg adjusted *P* value > 0.05).

Of note, antigens have a myriad of distinct functions, but the immune system only recognizes specific regions of the antigens—the epitopes. In some cases, epitope regions cover the entire antigen (as for *mpt64*), so selection acts on the antigen and epitope equally. In other cases, epitopes represent only a small fraction of the antigen and may be subject to different selective pressures than the rest of the gene (Fig. 2*C*). When exploring whether selection at the epitope level drives different antigen trajectories, we encountered the Rv1866 locus as a clear example. This antigen has a pN/pS value of greater than one near the root, but its value changes to less than one near the leaves, suggesting the action of distinct types of selection across the phylogeny; however, the epitopes contained are highly conserved with a pN/pS value of less than one during the complete trajectory.

In most cases (Dataset S3), the evolutionary trajectories of epitopes seem to be unlinked to the rest of the antigen, with most epitopes being conserved. We hypothesized that epitopes might reflect past selection events to adapt to different populations during the initial expansion of the MTBC. In general, the mean relative phylogenetic age (measured as the genetic distance to the root) of the nonsynonymous variants present in the epitopes is older than the nonsynonymous variants of the nonepitope regions of the antigen. This phenomenon can be observed when pooling all epitope vs. nonepitope variants (Welch *t* test, *P* value = 8e-07) and when splitting by different genes (Fig. 2*D*) (although with considerable overlap, as expected). Consequently, we expect fewer mutations to accumulate at phylogeny tips if epitope conservation becomes more important at a later stage. The proportion of mutations in epitopes falling in terminal branches (62%) is significantly lower than in sets of regions of the same size randomly selected from the nonepitope genome [70%, *z* score = −4.28, *P*(*x* < *Z*) = 0.00001] (Fig. 2*E*). This suggests the more robust nature of negative selection on epitopes than the rest of the genome in circulating strains.

Thus, our results provide further evidence for the generally unlinked nature of gene and epitope evolution, which had been previously established in smaller sets of samples (24, 38). In addition, we demonstrate that interaction with the immune system likely drives epitope conservation (as it is the only function in common among epitopes), while nonepitope regions reflect the selection of the gene's biological function. Finally, mutations in epitopes mainly reflect older fixation events, while the rest of the genome accumulates mutations more rapidly.

### Candidate Drug Resistance Regions Revealed by a Dataset Enriched for Multidrug-Resistant/Extensively Drug-Resistant Tuberculosis–Associated Strains.

**Fig. 3.**
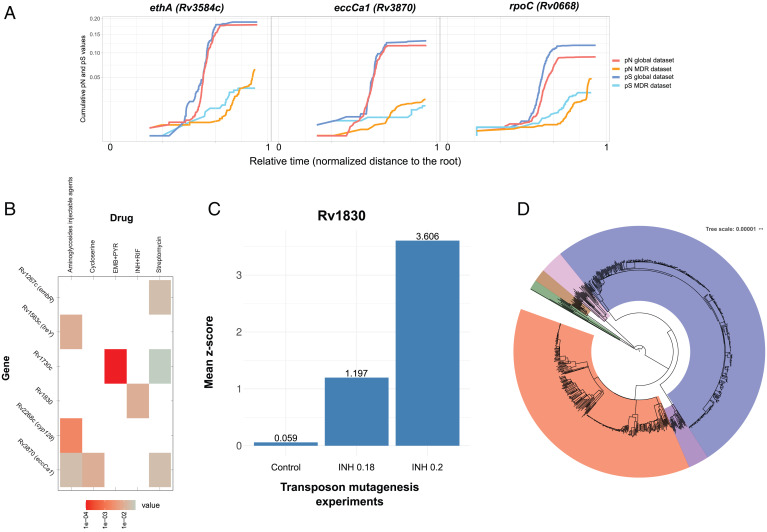
Identification of genes related to second-line antibiotic resistance. (*A*) Three genes showing signs of ongoing positive selection in the MDR-enriched dataset but ongoing purifying selection in the global dataset. The *x* axis represents the node to root genetic distance normalized in the zero to one range to merge data from both trees as a measure of relative time. The *y* axis represents the cumulative dN and dS values. (*B*) A computational model has been constructed for each antituberculosis drug to identify specific gene mutations associated with resistance. In the matrix, rows represent antibiotics, and columns represent genes suspected to be under positive selection in the MDR-enriched dataset. Colored cells (from gray to red) indicate a statistically significant association between nonsynonymous mutations found in the genes and resistant phenotypes. In this figure, only the less well–studied genes are shown. *SI Appendix*, Fig. S4 has the complete results. (*C*) Transposon sequencing (TN-seq) experiments have been performed (*x* axis) in the absence of isoniazid (control) and in two different isoniazid concentrations: 0.18 and 0.2 μg/mL. We observe a higher number of transposon insertions in Rv1830 compared with the mean number of insertions in the rest of the genome (calculated as a *z* score; *y* axis) in INH-treated experiments vs. control. This result provides *in vitro* evidence of how Rv1830 is a genetic determinant for isoniazid resistance. (*D*) Maximum likelihood phylogeny constructed with the MDR-enriched dataset showing an overrepresentation of L2 (blue) and L4 (red) strains. EMB, ethambutol; INH, isoniazid; PYR, pyrazinamide; RIF, rifampicin.

Identifying genes involved in resistance to second- and third-line drugs and new and repurposed drugs remains challenging. We reasoned that if our approach was powerful enough to identify changing selective pressures due to the introduction of first-line antibiotics, we should detect changes in genes associated with the treatment of multidrug-resistant (MDR) and extensively drug-resistant (XDR) tuberculosis patients. We assembled and compared a dataset enriched for MDR (*n* = 312) and XDR (*n* = 132) strains and additional sensitive controls to our global dataset (Dataset S1 and *SI Appendix*, Fig. S1*B*). Our analysis revealed instances of genes with an increased pN value toward the leaves of the tree for the MDR/XDR dataset compared with the global dataset. Our approach correctly identified genes associated with MDR, such as *gyrA* (quinolones), *ethA* (ethionamide), and *rpoC*, which compensate for the fitness cost of MDR strains (Fig. 3*A*). Importantly, we also identified less well–studied genes with a similar profile, including Rv0552, Rv1730c, *alr* (Rv3423c), *eccC4* (Rv3447), *eccCa1* (Rv3870) (Fig. 3*A*), and Rv3883c (*mycP1*). To formally evaluate their association to different drugs, we generated computational models (*Materials and Methods* and *SI Appendix*, Fig. S1*B*) to link the observed drug-resistant phenotypes with mutations in genes with a changing pN/pS pattern. Well-known resistance-conferring genes, such as *rpoB*, *katG*, or *rpsL*, exhibit a strong statistical association with drug-resistant phenotypes, as expected (*SI Appendix*, Table S1 and *SI Appendix*, Fig. S4). Corroborating our observations, the identified less well–studied genes displayed a significant association with resistant phenotypes for second-line drugs (Fig. 3*B*): for example, Rv1730c associated with first-line treatments (Wald test, *P* value = 0.04 for streptomycin and *P* value = 1e-4 for ethambutol + pyrazinamide), *treY* associated with aminoglycoside injectable agents (Wald test, *P* value = 0.01), Rv1830 associated with isoniazid and rifampicin (Wald test, *P* value = 0.01), *cyp128* associated with aminoglycosides (Wald test, *P* value = 0.002), and *eccCa1* associated with d-cycloserine and aminoglycoside injectable agents (Wald test, *P* value = 0.01 and *P* value = 0.02, respectively). In order to support our results on Rv1830, we obtained data from a transposon mutant library derived from H37Rv in our laboratory (43). The library was grown in two isoniazid concentrations (0.18 and 0.2 μg/mL), and the frequency of Rv1830 mutants in these antibiotic-selected experiments was compared with controls. We observed a mean of 1.2 and a 3.6-SD increase in the frequency of Rv1830 mutants, respectively (Wilcoxon's test, *P* value = 0.023 and *P* value = 0.001), supporting this gene's role in isoniazid resistance (Fig. 3*C*).

Of note, our analysis did have certain limitations; for example, given the combined therapy administered in tuberculosis treatment, the same gene may correlate with several antibiotics. Thus, the genes shown above may reflect selection for some other drugs. Likewise, given the enrichment of this dataset with L4 and L2 strains (Fig. 3*D*), nonsynonymous phylogenetic variants in genes, such as *fadD30* (Rv0404), *nrdZ* (Rv0570), Rv1825, *ephB* (Rv1938), and *glpQ1* (Rv3842c), appear to be associated with drug-resistant phenotypes but are likely neutral markers, a previously reported phenomenon (44). The identification of previously uncharacterized genes represents the overall value of the analysis, with links to individual drugs requiring corroboration by fine-grain *in vitro* experiments.

### Selection Also Acts in Noncoding Regions.

Beyond mutations affecting coding regions, we (and others) have established the importance of mutations in intergenic regions in shaping the pathogen's phenotype, as they can alter gene regulation. Hence, natural selection can also target these positions. Using a Poisson distribution, we identified 290 intergenic regions possessing more mutations than expected by chance (Benjamini-Hochberg adjusted *P* value < 0.05); 270 of the intergenic regions harbor homoplastic mutations, representing a good correlate of positive selection in the MTBC. Certain mutations had been previously categorized as resistance-conferring variants, including 1673425C > T (upstream *fabG1*), 4243221C > T (between *embC-embA*), or 2715342C > G (upstream *eis*) (Dataset S4). We found other mutations in intergenic regions suspected of being related to drug resistance; however, the exact mutations were not present in the PhyResSe and ReseqTB catalogs.

We also calculated the ratio of intergenic variants per intergenic site (pI) compared with the ratio of synonymous variants per synonymous site of the flanking genes (pI/pS) for each intergenic region as a measure of selective pressure, as previously proposed by Thorpe *et al.* (45). We found a mean pI/pS value of 1.03 (95% CI: 0.98 to 1.07), near the expected value of 1 when under no selection; however, 123 intergenic regions appeared as outliers of this distribution (Dataset S5) as they exhibit pI/pS values greater than 2.058 [calculated as Q3 + 1.5 × IQR (46)]. A gene set enrichment analysis of gene ontology (GO) functions of flanking genes of these intergenic regions demonstrated that the most overrepresented functions (hypergeometric test, Benjamini-Hochberg adjusted *P* value < 0.05) are responses to acid chemicals, oxidation-reduction (REDOX) processes, and regulation of DNA templated transcription. The identification of REDOX is in agreement with oxidative metabolism playing a role in macrophage survival and drug resistance (43, 47, 48). A previous study reported that changes in regulatory regions (mostly intergenic) could significantly affect the transcription rates of downstream genes (49). Therefore, the positive selection of these regions may not be surprising.

## Discussion

Pathogen diversity reflects a balance between evolutionary forces. In the case of the virtually clonal MTBC (9), highly diverse and highly conserved genes can be identified, despite low genetic diversity (1), thereby suggesting the activity of distinct evolutionary forces. While metrics, such as pN/pS, present with certain limitations (39), they allow the identification of the footprints of evolutionary forces. pN/pS has the power to identify selection at the genome-wide level (26, 27), including traces of positive selection in specific genes, gene categories, and/or lineages (23, 50, 51). Analyses revealed an average pN/pS value across the MTBC genome of around 0.7, well below the value of 1 expected for any organism but high compared with others. This likely reflects the recent emergence of the MTBC with the presence of many transitory polymorphisms (39) and the impact of genetic drift in the form of bottlenecks and founder effects (25). However, the balance of evolutionary forces shaping genetic diversity is dynamic, and what was under positive selection or drift in the past may be under negative selection in the present and vice versa. This idea is illustrated in our work by the striking discovery of scars of elevated nonsynonymous rates in almost half of the MTBC genome, contrasting with previous reports (23, 38).

Our analyses identified different temporal evolutionary dynamics in *M. tuberculosis* genes. In one important category, genes are subjected to positive selection or genetic drift early in MTBC evolution but to purifying selection near the leaves. A prominent example of this phenomenon is the accumulation of early nonsynonymous variants in epitopes, such as *mpt64*. Deep mutations may reflect past events, such as founder effects or drift, but our analysis suggests that mutations in epitopes are older when compared with other regions of the genome and that epitope evolution is not linked to the evolution of the rest of the antigen and functional category. These observations are compatible with scenarios suggesting early coevolution of host and pathogen populations (5).

We also identified genes subjected to purifying selection in the past but to current positive selection. The abrupt shift in the pN/pS values in resistance-conferring genes illustrates the impact of antibiotic treatments on MTBC evolution. While our approach detected an increase in the pN/pS in a set of genes in MDR and XDR strains, we did not observe this increase in strains not exposed to second-line drugs. This finding allowed for the proposal of a set of candidate genes that confer resistance to second-line antitubercular drugs. Previous reports have suggested that genes, such as Rv1830 or *eccCa1*, can confer resistance to MDR treatments (15, 52–56); however, genes identified in this study highlight our incomplete understanding of the genetic basis of resistance, in particular for second-line and new drugs. Our approach points to candidates that will require follow-up experiments. Nevertheless, our results are strongly affected by the fact that tuberculosis therapies consist of the administration of combined drugs, making it difficult to assign the signal detected on specific genes to single antibiotics. This problem could be alleviated after large collections of strains with associated phenotypes become available (57, 58) and with evolutionary approaches that can inform genetic association tests, like the one presented here

Our approach also detected genes unrelated to antibiotic resistance that have been subjected to recent positive selection, a finding missed when applying averaged pN/pS ratios. We commonly encountered the sensor component of 2CSs in this gene set, and our previous data established robust signs of recent positive selection in *phoR*, which encodes the sensor component of the PhoPR 2CS (9, 59). This finding suggested that nonsynonymous mutations in *phoR* participate in host adaptation by regulating PhoP, a major regulator of MTBC physiology and virulence. We now show a similar occurrence in two other sensor proteins—KdpD and NarS. Thus, the accumulation of nonsynonymous mutations in sensor proteins may represent a common strategy used by mycobacteria to adapt to the changing environment during infection.

In addition to coding regions, we also found traces of selection in noncoding sequences, which agrees with previous findings (45). While identifying selection pressures on intergenic regions remains challenging, given the problematic interpretation of the functional effect of variants that fall in these areas, homoplastic mutations and the comparison of variants against surrounding genes provide a good framework. Variant accumulation in these regions can impact the regulation of nearby gene expression (16, 49). Again, drug resistance appears to represent the strongest selective force; however, variants found in these regions also impact transcription factor activity and oxidative metabolism.

We are aware of the limitations of our current study. The study of past traces of selection in MTBC members remains challenging due to the low genetic diversity present; however, we attempted to maximize genetic diversity to gain resolution by including a broad representation of the main MTBC lineages. Unfortunately, subtle traces of selection affecting small subclades or groups of strains can be masked using this strategy; indeed, this is illustrated by our study when lineage-positive signs of selection fail to appear in our analysis. For example, Menardo *et al.* (41) have described a high number of nonsynonymous mutations in the epitopes of *esxH*. This finding is not reflected when considering all lineages but only when we search lineage by lineage (Dataset S3). Further analysis focusing on specific subclades, using thousands of strains, may illuminate differential evolutionary pressures within the MTBC. Furthermore, we only analyze mutations fixed in the phylogeny, so we only infer an approximate picture of the evolutionary forces that have shaped complex evolution in the past. In addition, the low variability present in the MTBC, strain subsampling, and lack of metadata/dates for most deposited genomes make absolute dating for some studied mutations extremely challenging. We have tried to date our dataset, and although our dating seems to make sense at least in drug-resistant genes (antibiotic selective pressure was not expected to appear after 1940), the fact is that the type of sampling that we have used in our analysis did not allow for generalization of the dating results obtained. Our initial interest was to detect global signs of selection in the whole MTBC. Thus, we reduced the original phylogeny by pruning closely related strains. Although this allowed us to computationally afford complex analysis, this led to an artificial enlargement of the tree terminal branches. Mutations mapped on long branches are difficult to date, as they have occurred at some point between the dates inferred for the defining nodes of a branch. Thus, while the overall shapes of the pN/pS trajectories do not change a lot, the exact point (date) at which changes in selection occurred cannot be properly estimated. Finally, in some cases, very recent selection signals may be confounded by transitory polymorphism (for example, when intrahost selection occurs), although the impact is expected to be minor in our dataset given the sampling strategy. We are also aware that, in some cases, genetic drift may be mistaken with other selection forces; however, this does not preclude those changes from having a functional effect (25).

Finally, we note that our approach can be used as a blueprint to study the evolution of several bacterial species. For example, the *Salmonella* genus includes strains exhibiting high host specificity and those with the general ability to infect many hosts (60). The gene by gene evaluation of past and current selective pressures could shed light on the genomic determinants that drive differing specificity. The same approach could be valid with *Helicobacter pylori*, a pathogenic bacteria that causes gastric infections and is highly specialized at infecting human hosts (61, 62). Therefore, our method could be a natural extension of the current population genomic pipelines in bacterial pathogens that are based on defining the pangenome for a set of strains, identifying recombinogenic regions, and building a “clonal” phylogeny after removing recombination. The MTBC displays virtually no recombination or ongoing horizontal gene transfer (which is not the case for *H. pylori* or *Salmonella*), making the interpretation of the results more straightforward; however, we anticipate that, taking into account population structure, our approach can be adapted to answer a range of evolutionary questions in pathogen evolution.

## Materials and Methods

### Variant Analysis Pipeline and Phylogenetic Reconstruction.

All samples were analyzed using our variant analysis pipeline, which has been extensively described in a previous publication (63). Briefly, FASTQ files were trimmed to remove low-quality reads using fastp (64) (version 0.12.5, arguments –cut_by_quality3, –cut_window_size = 10, –cut_mean_quality = 20, –length_required = 50, –correction) and aligned to the most likely inferred ancestor of the MTBC (24) using the BWA-MEM algorithm (65). Potential optical and PCR duplicates were removed with Picard tools (66), while reads with a mapping quality value (MAPQ) value of <60 were also discarded. Variant calling was performed using SAMtools (67), VarScan (68), and GATK (69). A pileup file was created with SAMtools from the BAM files, and VarScan was then used to extract variant positions from this pileup file (version 2.3.7, arguments –*P* value 0.01 –min-coverage 20 –min-reads2 20 –min-avg-qual 25 –min-strands2 2 –min-var-freq 0.90), while GATK was used to extract insertions and deletions (INDELS) (version 3.8-1-0-gf15c1c3ef, HaplotypeCaller and SelectVariants functions). To remove mapping errors, detected variants were discarded if found in INDEL areas or areas of high variant accumulation (more than three variants in a 10 base pairs defined window). Variants were then annotated using SnpEff (version 4.2) (70). Variants associated with proline–glutamate/proline–proline–glutamate (PE/PPE) genes, phages, or repeated sequences were also filtered out (Dataset S6) as they tend to accumulate false-positive SNPs owing to mapping errors. Finally, with the selected high-quality variant calls, a nonredundant variant list was created and used to retrieve the most likely allele at each genomic sequence to generate a variant alignment.

The first phylogeny was constructed with all samples that passed a minimum depth coverage threshold (median 25×) and had no mixed infections (*n* = 9,240). This initial phylogeny was constructed using MEGA-CC (71) and the neighbor-joining algorithm. Later, we pruned the phylogeny with Treemmer (72) to obtain a smaller tree for subsequent computational analyses. A reduction of just 5% of the initial genetic diversity led to the selection of 4,958 samples. With these selected samples, a maximum likelihood phylogeny was constructed using IQTREE (73) (version 1.6.10) with the general time reversible (GTR) model of evolution, taking into account the invariant sites, and with an ultrafast bootstrap (74) of 1,000 replicates.

### Phylogenetic Variant Mapping and pN/pS Trajectories.

After phylogenetic reconstruction, the mutations called in the 4,958 samples (*n* = 368,719) were mapped onto the phylogeny. For his purpose, the ancestral state of each polymorphism in each node was reconstructed using PAUP (75) with a weight matrix that punished reversions with a 20× multiplier. From this information, the phylogenetic branch at which each variant appeared was obtained. Later, a relative age derived from the branch-length information for each variant was assigned for each variant. This relative age is the genetic distance from the ancestral node to the bisection point of the target branch on which the variant appears. Finally, the cumulative pN (nonsynonymous variants/nonsynonymous sites) and pS (synonymous variants/synonymous sites) trajectories were calculated for each gene using the potential synonymous and nonsynonymous sites inferred using the SNAP tool (76) and plotting the cumulative pN, pS, and pN/pS values at each time point. For each time point (or distance to root point), we took into account all the variants that appeared before this time point. Later, we have represented the trajectories by connecting the different pN, pS, and pN/pS points of consecutive time points, hence obtaining a trajectory instead of a scatterplot.

We classified the genes in two big categories according to their pN/pS trajectories. On the one hand, we have genes that have been subjected to the same type of selection across all their phylogenetic history. On the other hand, we have those that have suffered from changing types of selection. For the first big group, those genes that had their pN/pS values under one (category 1) or above one (category 2) for almost all their evolutionary history (95% of the sampled points) have been classified as pS almost higher or pN almost higher. The second group, those having pN/pS values greater than one and less than one at different sampling points, includes a myriad of different scenarios. We were interested in those that were reflecting sharp changes in the action of selection: genes that seemed to evolve under purifying selection early in the phylogeny but later under positive selection and vice versa.

We focused on those trajectories that crossed the pN/pS = 1 limit an odd number of times, so the pN/pS will be different at the beginning of the trajectory and at the end (over or under 1). However, pN/pS trajectories that crossed that boundary five, seven, or more times mean that pN/pS was approximately one for a while, which could mean that change in selection was not sharp or that genetic drift was acting on this gene. So, depending on the direction of the trajectory (from pN/pS < 1 to pN/pS > 1 or the other way around) and the number of times that the trajectory crosses the pN/pS = 1 limit, we divide this second group into two other subgroups: genes in which pN > pS but inverts to pN < pS at a certain point (category 4) and genes in which pS > pN but inverts to pS < pN at a certain point (category 3). All other genes were classified as “variable” (category 5).

**Fig. 4.**
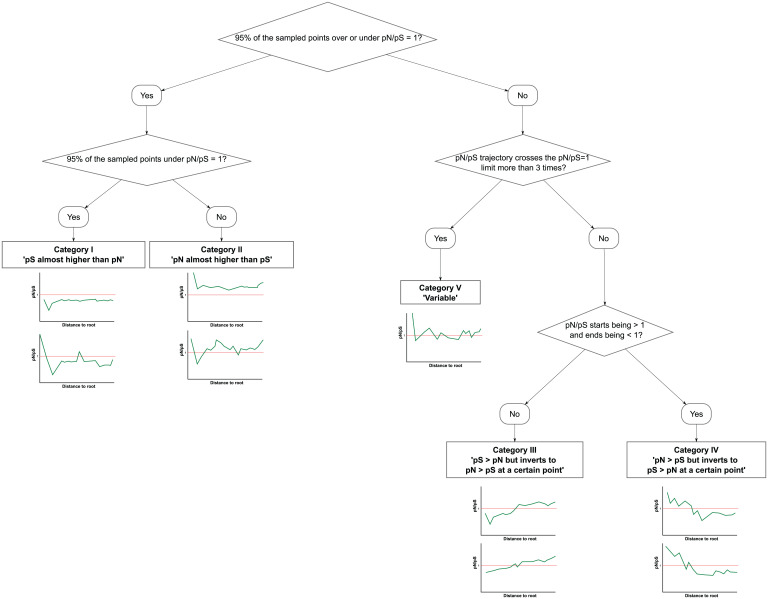
Schema of the algorithm followed for initial classification of the pN/pS trajectories.

So, briefly, we classified the genes according to their pN/pS trajectories with the following criteria (Fig. 4).

1) Genes with a cumulative pN/pS less than one at more than 95% of the sampled points were classified as pS almost always higher than pN.
2) Genes with a cumulative pN/pS greater than one at more than 95% of the sampled times were classified as pN almost always higher than pS.
3) Genes in which the cumulative pN/pS changed from greater than one to less than one or vice versa less than four times and the cumulative pN/pS started being less than one but ended greater than one were classified as pS > pN but inverts to pN > pS at a certain point.
4) Genes in which the cumulative pN/pS changed from greater than one to less than one or vice versa less than four times and the cumulative pN/pS started being greater than one but ended less than one were classified as pN > pS but inverts to pS > pN at a certain point.
5) Genes in which the cumulative pN/pS changed from greater than one to less than one or vice versa more than three times were classified as variable.

This classification was reviewed manually at a later stage. Genes with less than 10 mutations were not considered for subsequent analyses. All the trajectories calculated are provided in Dataset S7 (https://doi.org/10.6084/m9.figshare.19335854). In addition, all the SNPs called and the information associated with them are provided in Dataset S8.

We have also calculated the pN/pS trajectory for the complete genome by concatenating all the coding regions together and taking into account all the SNPs falling in these regions (*SI Appendix*, Fig. S3).

The cumulative pN/pS variation for each gene was calculated as

$$\text{pN/pS}\;\text{var}=\overset{n}{\underset{t=4}{\sum }}x_{t}-x_{t-1},$$

with *x* being the cumulative pN/pS value at each of the sampled *t* points. The first three values of each gene's cumulative pN/pS value were not considered, as the initial values can show significant differences due to a low number of mutations.

We have calculated two *z* scores from two different tests. First, the stability of the trajectories test is against the zero value (the expected cumulative pN/pS variation if selection was absolutely stable) to test if changing and stable selections were acting in the different categories. Second, the natural selection test is against the mean pN/pS value across time for the complete genome (in log scale) to see if the effect/direction of selection was different for the “stable” categories and the complete genome.

The *z* scores were calculated as

$$z\;\text{score}=\frac{x-\mu }{\sigma },$$

with *x* being either the zero value or the mean pN/pS value across time for the complete genome (in log scale), μ being the mean of the distribution for each category, and σ being the SD of the distribution for each category.

Mean pN/pS across time for each gene and for the complete genome were calculated as

$$\text{mean}\;\text{pN/pS}=\frac{\sum_{t=4}^{n}x_{t}}{n-4},$$

with *x* being the cumulative pN/pS value at each of the sampled *t* points. Again, the initial three values of each gene's cumulative pN/pS value were not considered.

### Epitope and Antigen Analysis.

All linear epitopes (*n* = 1,556) found in the Immune Epitope Database (IEDB) (42) that belonged to *M. tuberculosis* in August 2019 were downloaded. All linear epitopes with overlapping coordinates with regard to the H37Rv reference strain were merged into unique nonoverlapping “contigs” (*n* = 718). The potential synonymous and nonsynonymous sites were inferred using the SNAP tool (76). All genes containing such epitopes were considered antigens, except those genes not considered in the variant calling step, as explained above (PE/PPE, phages).

The percentage of SNPs that occur in these 718 regions that appear in terminal branches of the phylogeny was determined using the information derived from PAUP. The percentage of SNPs in the rest of the genome (not considering these 718 regions) that fall in terminal branches was also determined. To evaluate if the difference between these values was statistically significant, 718 segments of the nonepitope genome with the same length as the epitope regions set, 1,000 times, were selected. For each iteration, the percentage of SNPs found in terminal branches was calculated and plotted in a distribution. Finally, a *z* score (the formula is given above) between the distribution and the value observed for the epitopes was calculated.

### Gene Set Enrichment Analysis.

Several approaches for functional category enrichment were performed to compare genes present in our sets of interest against other genes. For the essentiality enrichment, the *in vivo* (77) and *in vitro* (78) classifications of genes were used, and the enrichment in these categories was tested with Fisher tests. For GO enrichment, the Bingo tool (79) was used with a hypergeometric test (sampling without replacement) and the Benjamini–Hochberg correction for multiple testing comparisons. Finally, the enrichment of the functional categories was also evaluated (80), employing Fisher tests corrected with the Benjamini–Hochberg procedure.

### Dating Analysis.

In order to estimate the time tree of our dataset, we have split the complete phylogeny into 18 reduced datasets (L1.1.1, L1.1.2, L1.1.3, L1.2.2, L2, L3, L4.1.1, L4.1.2, L4.2.1, L4.2.2, L4.3.1, L4.3.2, L4.3.3, L4.3.4, L4.4, L4.5, L4.6, L4.10). We implemented a coalescent Bayesian constant growth model available in Beast 2.6 (81) with the GTR + Γ model of nucleotide substitution, with  Γ = 4. We used an exponential distribution (M = 1) for the effective population size tree prior. Since dates were unavailable for most samples, we fixed the strict clock using the values proposed by Menardo *et al.* (82) for each lineage. Invariant positions were specified in the xml files following ref. 83. Parameters were estimated using Markov chain Monte Carlo (MCMC) Bayesian inference with 1 × 107-step-long chains with the exception of the larger datasets (L2, L3, L4.1.2, L4.3.4, L4.10), for which longer chains were run (1 × 108) and the tree topology was fixed. In all cases, a total of 105 steps were sampled in the log files, and the initial 10% of the MCMC was removed as burn-in. Adequate mixing of parameters was assessed using Tracer v.1.7.1 (84) by verifying that each parameter reached an effective sampling size above 200 and that traces showed stationarity and good mixing. The final posterior distribution contained a total of 9,000 trees, annotated with Treeannotator v.2.6.3 and visualized in FigTree v.1.4.3 (85).

Next, we have assigned an age to each mutation. Following the same approach as in the distance to root value, for each mutation the age was calculated as the middle value between the inferred ages of the branch-defining nodes in which the mutation was mapped. Once the mutations have been dated, we have calculated new pN/pS trajectories using the inferred age instead of the relative dating derived from the distance to root value.

### Drug-Resistant Dataset Preparation and Analysis.

Drug-resistant strains were downloaded from the TBportals database (86) on 22 October 2019 (*n* = 656). Samples were classified according to their drug-resistant phenotype and then passed through the variant analysis pipeline described above. A maximum likelihood phylogeny was constructed using IQTREE with the previously described options, including samples from the Comas *et al.* (10) study to achieve nodes from lineages underrepresented in the TBportals database.

The pN/pS trajectories were calculated and classified as explained for the other dataset.

A matrix was next created that included, for each sample, phenotypic information for each tested drug (resistant/susceptible) and the presence/absence (one/zero) of nonsynonymous mutations in the gene set classified as having a trajectory in which the pS > pN but inverts to pN > pS at a certain point for each sample. These nonsynonymous mutations were those that appeared after the point at which the pN/pS increases over one. The matrix has the form shown in Table 1.

**Table 1.** Schema of the data matrix used for regression models construction

Sample	INH + RIF	EMB + PYR	…	*rpoB*	*katG*	*gyrA*	…
1	R	R	…	1	1	1	…
2	R	S	…	1	0	0	…
…							

This matrix has the phenotypic information for each tested drug codified as R (resistant) or S (suceptible). It has also genotypic information for the specified gene set codified as 1 (presence of nosynonymous mutations after the pN/pS > 1 cross-point) or 0 (absence of nosynonymous mutations after the pN/pS > 1 cross-point). EMB, ethambutol; INH, isoniazid; PYR, pyrazinamide; RIF, rifampicin.

A set of binomial logistic regression models was constructed using this matrix, explaining the observed phenotypes based on the presence of nonsynonymous mutations on selected genes. Models were constructed using the glm() function in R, specifying the binomial distribution and the logit link. They were constructed as

model=glm (DRUG∼gene1+gene2+gene3+  …, data=MATRIX, family=binomial(link=logit)).

These models were trimmed a posteriori following a backward stepwise methodology using the step() function, selecting the set of regressors that show the best Akaike information criterion. Later, the summary.glm() function was applied on the trimmed models to obtain the *P* values of each coefficient.

### Transposon Sequencing Experiment on Rv1830.

We used TN-seq data described in Furió *et al.* (43) to further confirm the role of Rv1830 in isoniazid resistance. Briefly, a saturated transposon insertion library was generated and grown in the presence and absence of isoniazid (two concentrations, 0.18 and 0.2 μg/mL, close to the minimum inhibitory concentration for this drug). Bacterial populations for each condition were sequenced, and insertions were mapped to each genomic feature to assess their importance in the context of resistance. Rv1830 showed a significant insertion increase in isoniazid-treated experiments of up to 3.6 SDs when compared with the genomic average.

## Supplementary Material

Supplementary File

Supplementary File

Supplementary File

Supplementary File

Supplementary File

Supplementary File

Supplementary File

Supplementary File

## Data Availability

Plots of all trajectories calculated (Dataset S7) have been deposited in Figshare (https://doi.org/10.6084/m9.figshare.19335854). Previously published data were also used for this work [data from Brites *et al.* (4), Coll *et al.* (29), Stucki *et al.* (30), Guerra-Assunção *et al.* (31), Zignol *et al.* (32), Bos *et al.* (33), Ates *et al.* (34), Comas *et al.* (10, 35), Borrell *et al.* (36), and Cancino-Muñoz *et al.* (37)].
