# Supplementary material for: Gene evolutionary trajectories in Mycobacterium tuberculosis reveal temporal signs of selection
Source: Proc Natl Acad Sci U S A. 2022 Apr 22;119(17):e2113600119. doi: 10.1073/pnas.2113600119 (PMC9173582; doi:10.1073/pnas.2113600119)
Supplement: Supplementary File [file pnas.2113600119.sapp.pdf]

PNAS
www.pnas.org

Supplementary Information for
Gene Evolutionary Trajectories in *M. tuberculosis* Reveal Temporal Signs of Selection

Álvaro Chiner-Oms[1,], Mariana G. López[1], Miguel Moreno-Molina[1], Victoria Furió[1], Iñaki Comas[1,2,*]*

1. Instituto de Biomedicina de Valencia, IBV-CSIC, Valencia, Spain
2. CIBER en Epidemiología y Salud Pública, Valencia, Spain

Email: achiner@ibv.csic.es (Álvaro Chiner-Oms), icomas@ibv.csic.es (Iñaki Comas)

This PDF file includes:

Figures S1 to S4
Table S1
Legend for Datasets S1 to S8
SI References

Other supplementary materials for this manuscript include the following:

Datasets S1 to S8

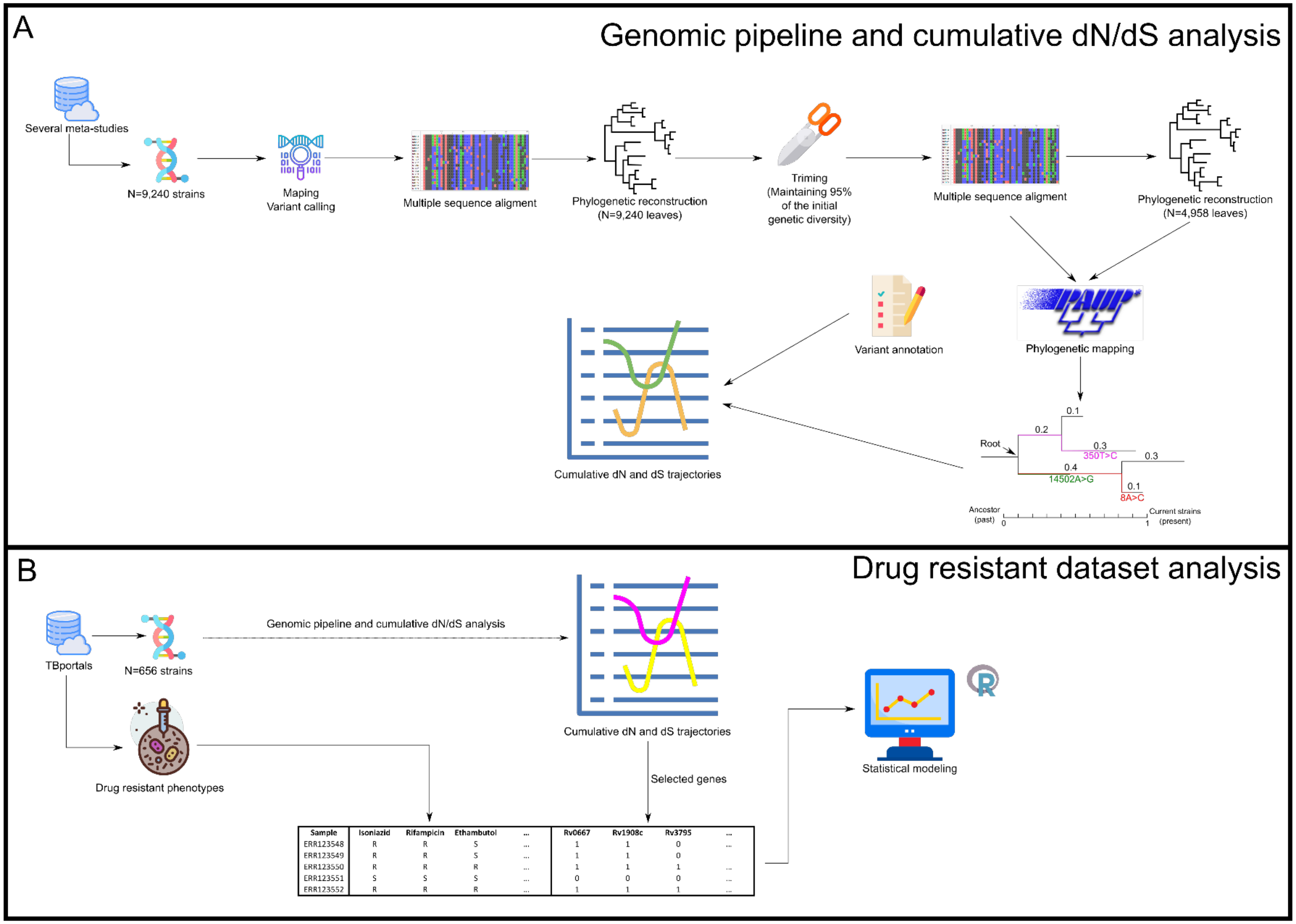

Figure S1. Workflow Followed in Different Analyses. a. From public repositories, we downloaded more than 9,000 MTBC genomes. After reconstructing a phylogenetic tree, the dataset underwent a trimming process to reduce the number of samples while maintaining as much genetic diversity as possible. From these reduced datasets, we reconstructed a tree and an alignment. PAUP mapped each detected polymorphism into the phylogeny. Finally, knowing the annotation of the polymorphisms and the branch in which they appeared allowed us to generate pN/pS trajectories. **b.** TBportals was used to obtain a dataset enriched for resistant strains. The same approach as described above was applied (except for the trimming step), thereby obtaining pN/pS trajectories for each gene based on the information of this new dataset. We also downloaded drug-susceptibility test (DST) information for each resistant strain. Combining both the genomic and the phenotypic information allowed the generation of computational models linking the observed phenotypes to mutations in specific genes.

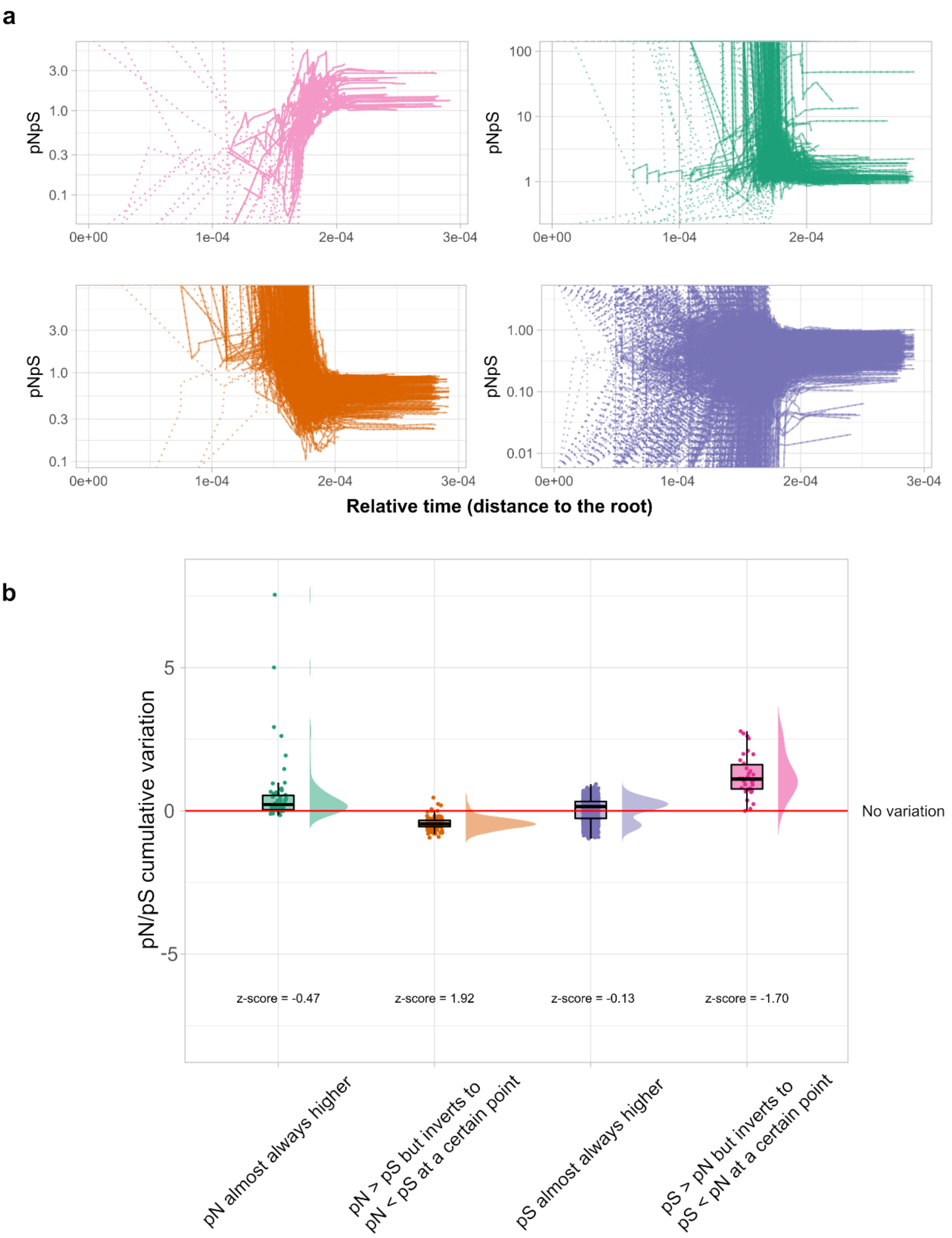

Figure S2. Classification of Genes According to pN/pS Trajectory. a. pN/pS variation across the phylogeny, from root to tips. Each line corresponds to a different gene. Genes were classified as: (i) pS almost always higher than pN (blue); (ii) pN almost always higher than pS (green); (iii) pS > pN but inverts to pN > pS at a certain point (pink); (iv) pN > pS but inverts to pS > pN at a certain point (orange); (v) pN and pS had complex trajectories (not plotted). The red horizontal line marks pN/pS = 1. The first three values of the trajectory (dashed in the plots) were not considered for classification, and the rest of the analysis as they present with high variability due to a small number of mutations. **b.** Cumulative pN/pS variation distribution for each gene category. Categories reflecting 'stable' trajectories ('pN almost always higher' and 'pS almost always higher') accumulated low variance in pN/pS and displayed no significant differences (Welch t-test, p-value > 0.05). In both cases, the pN/pS cumulative variation is around zero. In contrast, categories with changing trajectories display significant differences (Welch t-test, p-value << 0.01), using 'pS almost always higher category' as the reference category.

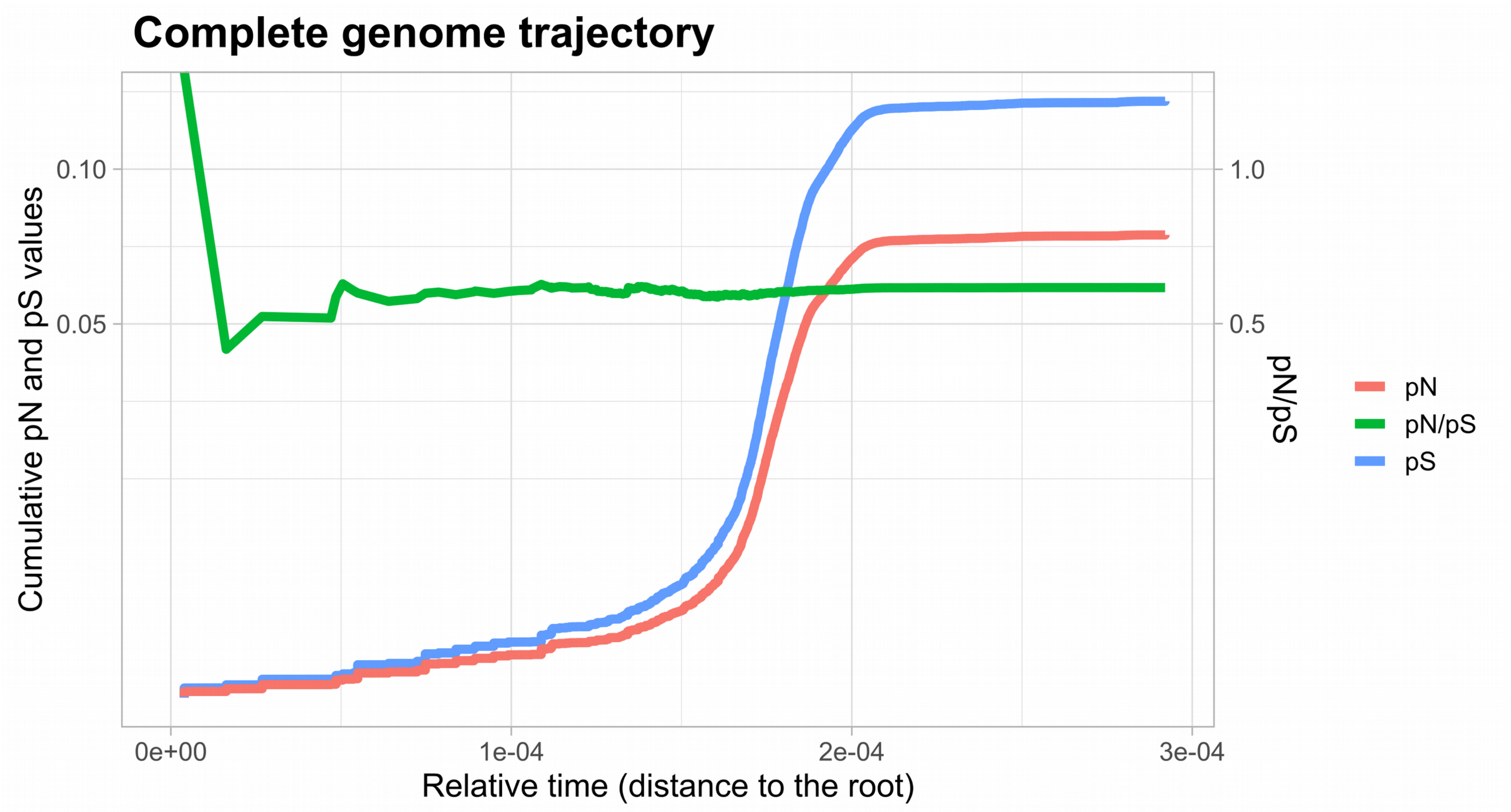

Figure S3. Cumulative pN, pS and pN/pS trajectories for the complete genome

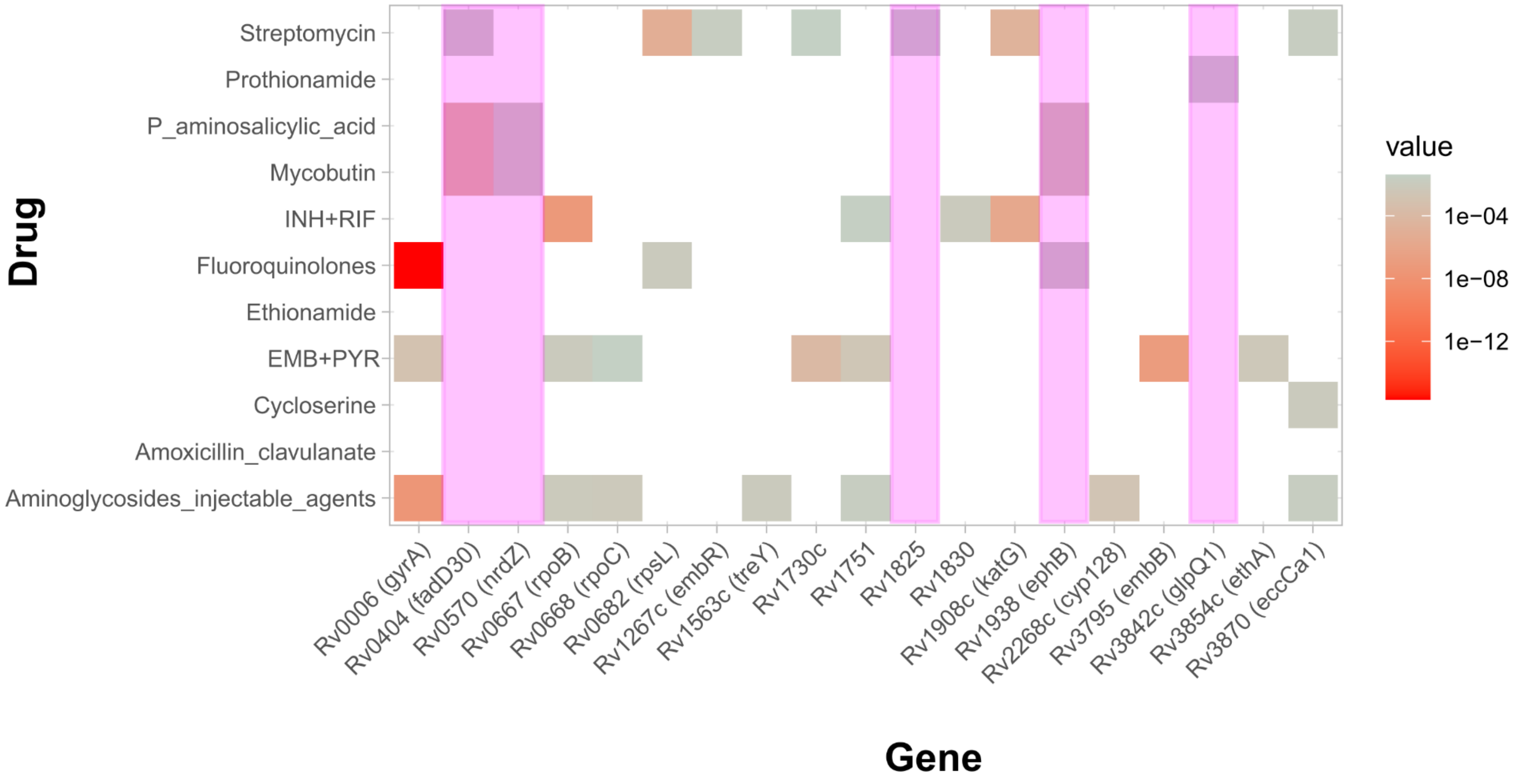

Figure S4. A computational model has been constructed for each antituberculosis drug to identify specific gene mutations associated with resistance. In the matrix, rows represent antibiotics and columns represent genes suspected to be under positive selection in the MDR-enriched dataset. Colored cells (from gray to red) indicate a statistically significant association between non-synonymous mutations found in the genes and resistant phenotypes. Genes marked in pink show a strong association with drug-resistant phenotypes due to phylogenetic variants, suggesting that the association may be spurious.

	Rv0006 (*gyrA*)	Rv0404 (*fadD30*)	Rv0570 (*nrdZ*)	Rv0667 (*rpoB*)	Rv0668 (*rpoC*)	Rv0682 (*rpsL*)	Rv1267c (*embR*)	Rv1563c (*treY*)	Rv1730c
**INH+RIF**				4.29E-08					
**EMB+PYR**	1.00E-03			1.00E-02	4.00E-02				1.00E-04
**Aminoglycosides injectable agents**	2.48E-08			8.00E-03	7.00E-03			1.00E-02	
**Streptomycin**		2.00E-02				6.87E-06	2.00E-02		4.00E-02
**Prothionamide**									
**Para-aminosalicylic acid**		2.98E-05	8.00E-03						
**Mycobutin**		2.98E-05	8.00E-03						
**Fluoroquinolones**	2.00E-16					1.00E-02			
**Ethionamide**									
**Cycloserine**									
**Amoxicillin-clavulanate**									

	Rv1751	Rv1825	Rv1830	Rv1908c (*katG*)	Rv1938 (*ephB*)	Rv2268c (*cyp128*)	Rv3795 (*embB*)	Rv3842c (*glpQ1*)	Rv3854c (*ethA*)	Rv3870 (*eccCa1*)
**INH+RIF**	3.00E-02		1.00E-02	1.43E-06						
**EMB+PYR**	3.00E-03						9.54E-08		5.00E-03	
**Aminoglycosides injectable agents**	2.00E-02					2.00E-03				2.00E-02
**Streptomycin**		4.00E-02		2.22E-05						2.00E-02
**Prothionamide**								4.00E-02		
**Para-aminosalicylic acid**					2.00E-03					
**Mycobutin**					2.00E-03					
**Fluoroquinolones**					2.00E-02					
**Ethionamide**										
**Cycloserine**										1.00E-02
**Amoxicillin-clavulanate**										

Table S1. P-values (Wald t-test) of the computational models generated - Genes marked in yellow display significant values, but are probably due to phylogenetic markers.

Dataset S1 (separate file). Samples used in the analyses, including accession numbers and the main phylogenetic lineage

Dataset S2 (separate file). Classification of genes in the five main categories defined in the main results

Dataset S3 (separate file). Classification of the antigens/epitopes studied, including the categories proposed for each of the features and the lineages in which they show differential trajectories.

Dataset S4 (separate file). Homoplastic variants called in the intergenic regions analyzed.

Dataset S5 (separate file). 123 intergenic regions that exhibited pI/pS values that are outliers of the genomic pI/pS distribution. Observed and expected mutations in the intergenic regions, probability of observing SNPs by chance (Poisson distribution), and the pI/pS calculated are shown.

Dataset S6 (separate file). Genomic regions not considered for analysis.

Dataset S7 (separate file). Plots of all pN, pS and pN/pS trajectories calculated for the complete MTBC genome, for the antigen-epitope pairs (MTBC wide) and also for the antigen-epitope pairs but splitted by the main MTBC phylogenetic lineages (L1, L2, L3, L4, L5, L6, L7 and the animal-adapted strains). File can be found at https://dx.doi.org/ 10.6084/m9.figshare.19335854 (1).

Dataset S8 (separate file). All the nucleotide substitutions called in the genes, including the type of mutation, distance to the root, annotation, cumulative pN, pS and pN/pS and the phylogenetic lineage in which the mutations have been identified.

SI References

1. Chiner-Oms Á, Mariana G López, Miguel Moreno-Molina, Victoria Furió, Iñaki Comas. Data "Plots of all pN/pS trajectories calculated". Figshare. Available at https://dx.doi.org/10.6084/m9.figshare.19335854. Deposited 10 March 2022
